# N-doped reduced graphene oxide for room-temperature NO gas sensors

**DOI:** 10.1038/s41598-021-99883-9

**Published:** 2021-10-20

**Authors:** Yu-Sung Chang, Feng-Kuan Chen, Du-Cheng Tsai, Bing-Hau Kuo, Fuh-Sheng Shieu

**Affiliations:** Department of Materials Science and Engineering, National Chung Hsing University, Taichung, 40227 Taiwan, Republic of China

**Keywords:** Materials science, Nanoscience and technology

## Abstract

In this study, we use nitrogen-doped to improving the gas-sensing properties of reduced graphene oxide. Graphene oxide was prepared according to a modified Hummers’ method and then nitrogen-doped reduced graphene oxide (N-rGO) was synthesized by a hydrothermal method using graphene oxide and NH_4_OH as precursors. The rGO is flat and smooth with a sheet-like morphology while the N-rGO exhibits folded morphology. This type of folding of the surface morphology can increase the gas sensitivity. The N-rGO and the rGO sensors showed n-type and p-type semiconducting behaviors in ambient conditions, respectively, and were responsive to low concentrations of NO gases (< 1000 ppb) at room temperature. The gas-sensing results showed that the N-rGO sensors could detect NO gas at concentrations as low as 400 ppb. The sensitivity of the N-rGO sensor to 1000 ppb NO (1.7) is much better than that of the rGO sensor (0.012). Compared with pure rGO, N-rGO exhibited a higher sensitivity and excellent reproducibility.

## Introduction

Gas sensors are very important for a wide range of applications, including detection of harmful chemical vapors, industrial process control, and chemical processing plants^1,2^. The development of gas sensors is a highly critical research area that involves issues related to safety, health, and environmental risks^3,4^. Typical gas sensors use metal oxide materials, such as zinc oxide and tin oxide^5,6^. Metal oxide-based gas sensors operate at high temperatures (200–400 °C), which results in excessive consumption of energy and limits their long-term stability. Thus, it is important to develop new gas sensor concepts that can overcome these drawbacks.

In recent years, carbon-based nanomaterials, such as carbon nanotubes and graphene, have been used for gas detection at room temperature. Various gases have been tested using carbon nanotubes gas sensors^7–9^. However, these sensors show low sensitivity and long response time, or poor reproducibility, depending on the assembly process and the purity. Graphene-based materials are useful for the fabrication of high-performance sensors, especially for detecting gases at low operating temperatures. Graphene exhibits two excellent properties for gas detection: (1) High carrier mobility (200,000 cm^2^/V s) and high surface area (2630 m^2^/g), facilitating detection of resistance change during the process^10^ and (2) Low electrical resistivity (1.0 × 10^–6^ Ω cm) for detecting resistance change after adsorption or desorption and increase or decrease in target gas concentration^11^. However, pure graphene is intrinsically inert, while graphene oxide (GO) has multitudinous oxygen functional groups and is too insulating for fabrication of resistance-based sensors. Recently, several graphene-based composites have been reported for gas sensors. For example, Ganhua et al.^12^ prepared graphene directly from GO through one-step heating (200 °C, 2 h), which was responsive to low-concentration NO_2_ and NH_3_ gases in air at room temperature with excellent sensitivity. Jianwei et al.^13^ synthesized graphene/Pt with sensitivities of 14% (7%), 8% (5%), and 10% (8%), for 1000 ppm H_2_, NH_3_, and NO gases, respectively with (without) Pt nanoparticles at room temperature. Vardan et al.^14^ prepared a hybrid structure of reduced graphene oxide (rGO) and ZnO nanostructures exhibiting 40–50% better response to NO_2_ and H_2_ compared to pure ZnO sensors at an operating temperature of 200 °C. Hao et al.^15^ synthesized rGO/SnO_2_/Au hybrid nanomaterials through a one-step hydrothermal process, exhibiting a much better response/recovery time to 5 ppm NO_2_ compared with pure rGO and rGO/SnO_2_ at an optimal operating temperature of 50 °C. Sen et al.^16^ synthesized rGO/ZnO/Au hybrids through the wet chemical method with high sensitivity and fast response for detection of NO_2_ at an operating temperature of 80 °C. The improved sensor performance reported in the above studies is attributed to the incorporation of graphene/RGO-based nanocrystals/nanoparticles. However, their synthesis methods are complicated, requiring a high operating temperature and incorporation of precious metal or metal-oxide, and the sensor has a low response to low concentrations of gases. Therefore, reducing operating temperature and cost, and developing an environment-friendly strategy is very important to extend the applications of graphene-based gas sensors.

We prepared nitrogen doped rGO gas sensors with fast response and high sensitivity. Nitrogen-doped rGO (N-rGO) nanosheets were prepared directly from GO through the hydrothermal treatment in an ammonia solution. In the gas sensing process, N-rGO and rGO exhibited n-type and p-type semiconductor behaviors, respectively. We assessed the sensing characteristics of N-rGO at various concentrations of NO gas at room temperature.

## Materials and methods

### Chemicals

All chemicals were of analytic grade and used without further purification. Graphite powder (Choneye Pure Chemicals, Taipei, Taiwan) was used to prepare GO. Sodium hydroxide (NaOH; Echo Chemical, Miaoli, Taiwan) and an ammonia solution (Choneye Pure Chemicals, Taipei, Taiwan) were used to prepare rGO and N-rGO, respectively. The NO calibration gas cylinders (1000 ppb) used for the sensing studies were obtained from Air Products San Fu Co. Ltd., Taiwan.

### Preparation of GO

The modified Hummers’ method^17^ is used to prepared graphene oxide powders. The mixture of 2 g of graphite powder and 0.6 g of NaNO_3_ is mixed in 20 mL of concentrated H_2_SO_4_ solution under 30-min stirring in an ice-bath surrounding. Then, 10 g of KMnO_4_ was added slowly in the mixture solution which was stirred at 35 °C for 3 h. Follow, the mixture solution with 180 mL DI water was heated at 98 °C for 2 h. In order to eliminate the excess of KMnO_4_, 20 mL of 30% H_2_O_2_ was dropped slowly to the solution. Finally, the mixture solution was centrifuged and washed with 5% HCl and DI water to remove the metal ions and control the pH value to 7. The resulting solid was dried in a vacuum oven under a pressure of 101.32 Pa at 70 °C for 12 h.

### Preparation of rGO and N-rGO

Hydrothermal method is used to synthesize N-doped graphene from the raw material of graphene oxide. Dispersion of GO (0.05 g) in 50 mL of DI water is obtained by ultra-sonication for 2 h. Then, 20 mL of ammonia solution (25 wt.% in water) was added in dispersed solution. The Teflon-lined autoclave which contain the mixture solution is placed in drying oven at 200 °C for 24 h. The reacted solution from the autoclave which is cooled naturally to room temperature are centrifuged and washed several times with distilled water and ethanol after. The remaining mixtures are drying in a vacuum oven at 80 °C for 24 h. For fabrication of rGO powders, the NaOH solution is conducted in the same process instead of ammonia solution.

### Characterization

The optical properties were measured using a Shimadzu UV 3600 spectrophotometer at wavelengths in the range of 200–800 nm. The morphology and the dispersion level of rGO and N-rGO were analyzed by transmission electron microscopy (TEM, JEOL 200CX), field-emission scanning electron microscopy (FESEM, JEOL JSM-6700F), and scanning electron microscopy (SEM, Hitachi TM-1000). Raman spectra were recorded on an Andor DU401-BV multichannel confocal microspectrometer with 632-nm laser excitation. X-ray photoelectron spectroscopy (XPS, PHI 5000 VersaProbe) was performed using monochromated Al Kα radiation (1486.8 eV). All these measurements were carried out at room temperature.

### Fabrication of sensor and gas-sensing measurements

**Figure 1:**
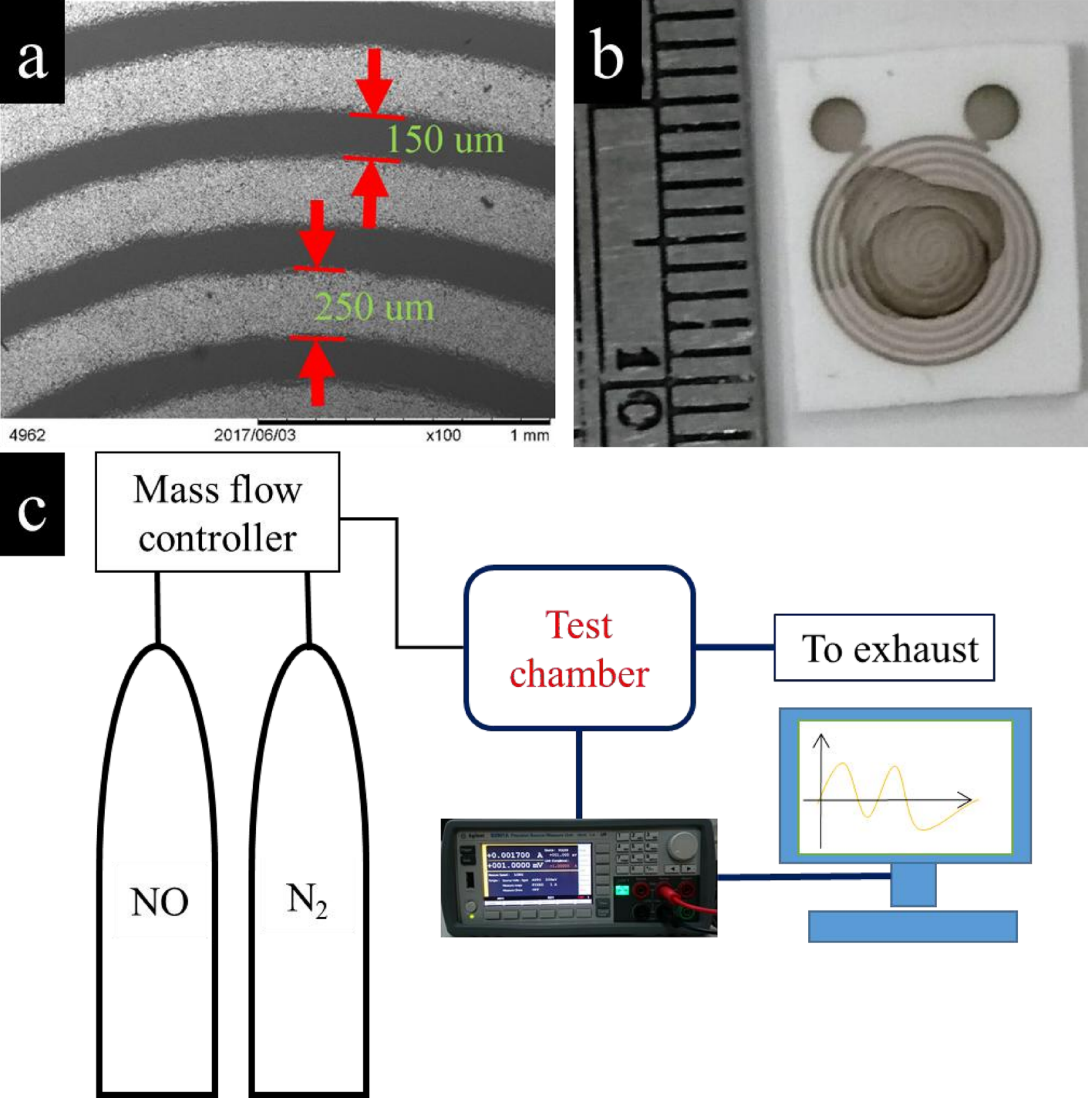
(**a**,**b**) Microelectrodes with an interdigital structure consisting of Ag–Pd conducting paths on an Al_2_O_3_ substrate. (**c**) Schematic of gas-sensing.

The sensor was fabricated using a three-layer configuration: sensing materials, Ag–Pd electrodes, and Al_2_O_3_ substrate. Figure 1a shows the SEM image of Ag–Pd electrodes. The prepared N-rGO was added to a 1:1 mixture of DI water and ethanol and then sonicated for 20 min. Then, a droplet was placed on a planar resistive-type sensor device which was then placed on a hotplate for 20 min to dry (Fig. 1b). Figure 1c shows the testing apparatus used for gas sensing. All the measurement results at room temperature were acquired by recording the corresponding electrical responses. The gas concentration was controlled by a mass flow controller (MFC, Brooks 5850E). The electrical resistances of the sensors were measured using a Keysight B2901A data acquisition system and collected in real time using a PC equipped with the corresponding data acquisition hardware and software. The desired concentration of the target gas was obtained by adjusting the flow rates with the MFCs, while maintaining a total constant flow rate of 200 sccm (mL/min). The response upon gas exposure was evaluated by measuring the electric current variation of the sensor using a bias voltage of 1 V.

## Results and discussion

### Structural and chemical characterization

**Figure 2:**
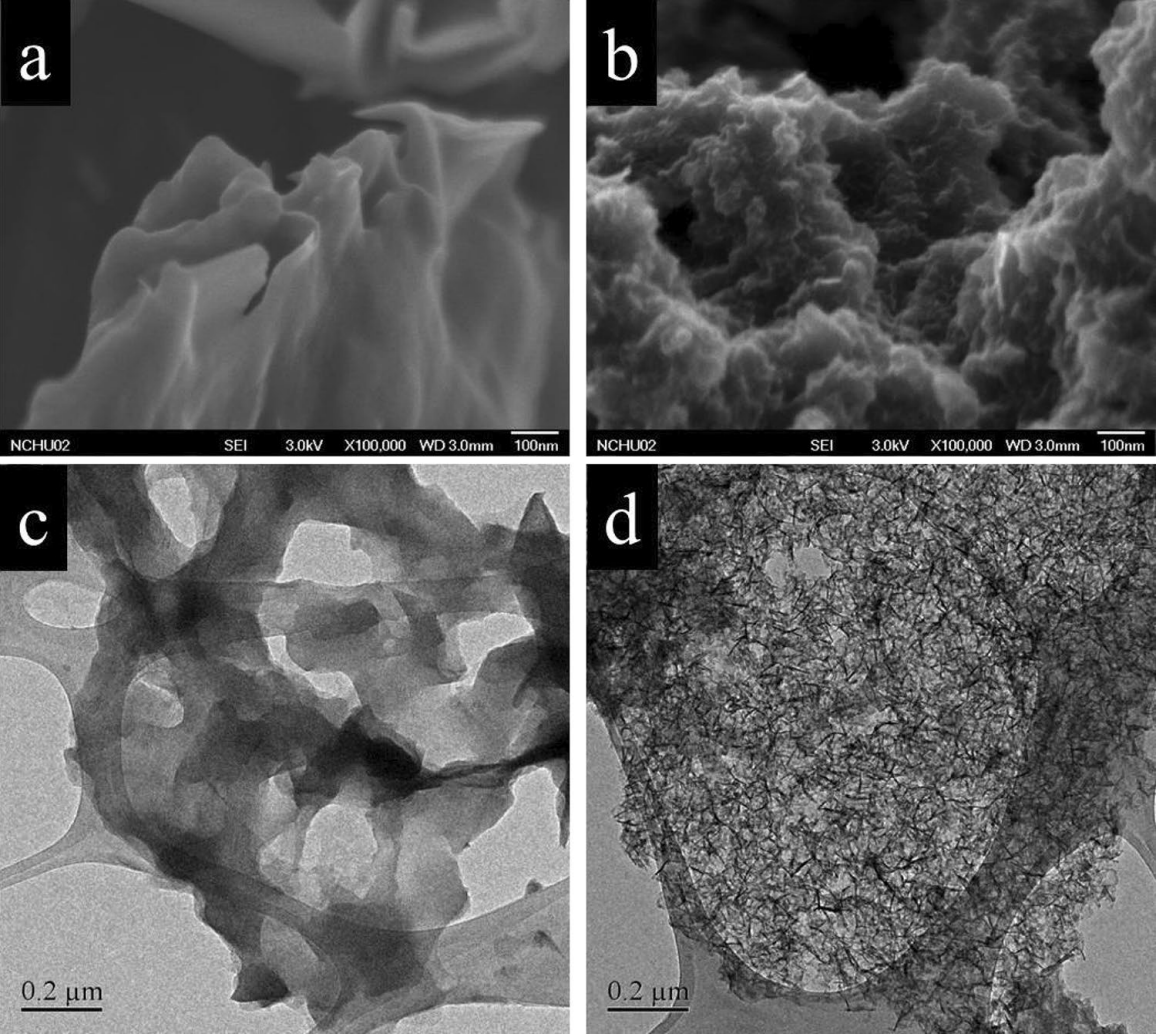
FESEM images of (**a**) rGO and (**b**) N-rGO; TEM images of (**c**) rGO and (**d**) N-rGO.

Figure 2a,b show the FESEM images of rGO and N-rGO, respectively. A smooth and sheet-like surface of the rGO sample is apparent in Fig. 2a. Figure 2b shows that N-rGO exhibits a folded morphology after the hydrothermal treatment. The TEM images of the rGO sample are shown in Fig. 2c, while those of the N-rGO sample are shown in Fig. 2d. It is evident that rGO is flat, transparent, and smooth with a sheet-like morphology and lateral dimensions ranging from 1 to 10 μm (Fig. 2c). On the other hand, the morphology of N-rGO resembles crumpled silk; this type of folding of the surface morphology can increase the gas sensitivity^18,19^.

**Figure 3:**
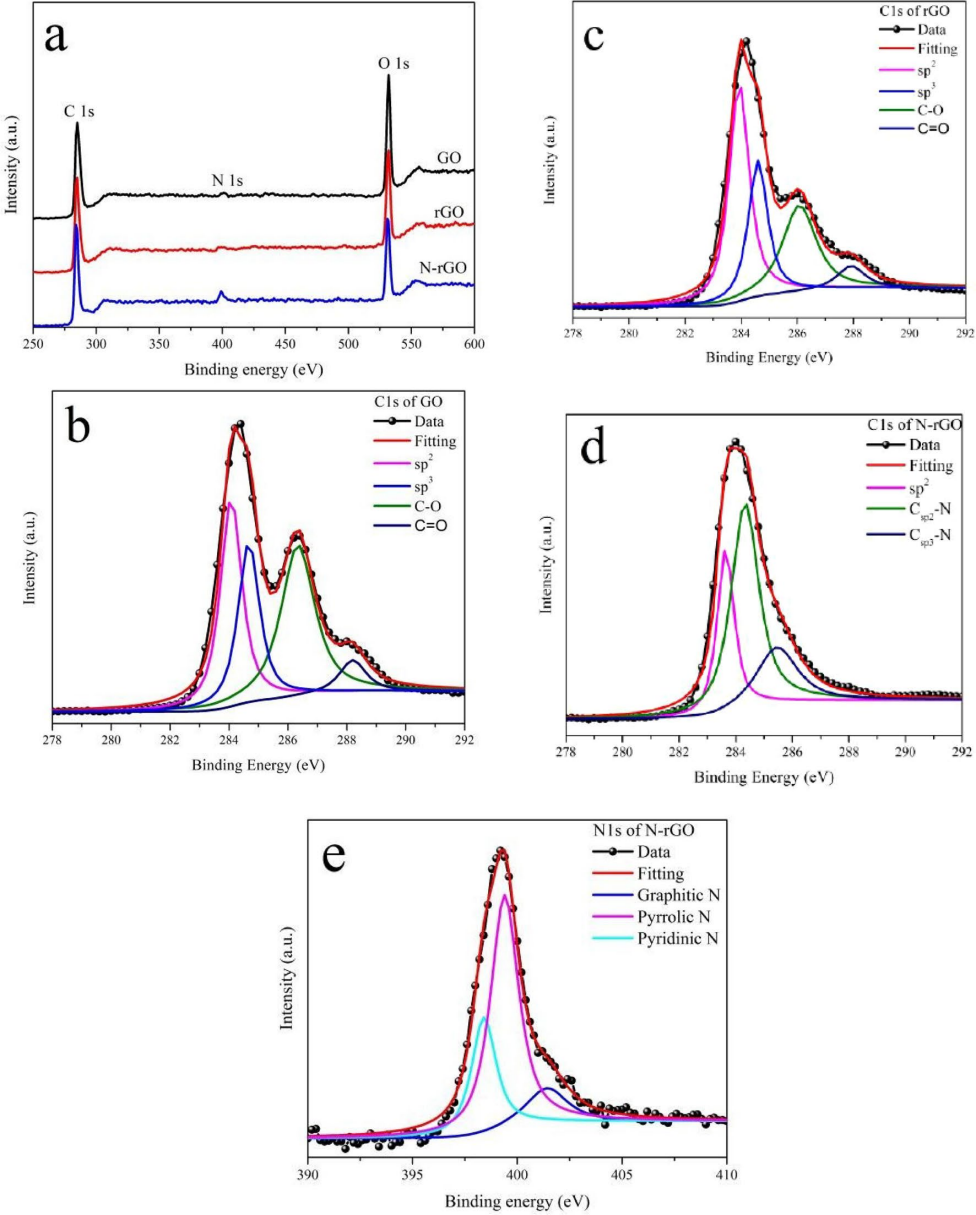
(**a**) XPS spectra of GO, rGO, and N-rGO; (**b**) C1s peak of GO; (**c**) C1s peak of rGO; (**d**) C1s peak of N-rGO; (**e**) N1s peak of N-rGO.

The main application of XPS is determination of binding energy of electrons for qualitative analysis of surface elements. Figure 3a shows the XPS spectra of the GO, rGO, and N-rGO samples; the major peaks correspond to C, N, and O elements, suggesting absence of impurities. Figure 3b,c show the C 1 s spectra of GO and rGO, respectively, wherein the major binding energy contributions are assigned to sp^2^ (C=C), sp^3^ (C–H), C–O (hydroxyl, epoxide, ether etc.), and C=O (carboxylic) bonds^20,21^. The intensity of the sp^2^ peak increases and that of the oxygen-related peaks decreases, further confirming the reduction of GO. Figure 3d shows the C 1s spectra of N-rGO, wherein the major binding energy contributions are assigned to sp^2^ (C=C), Csp^2^-N, and Csp^3^-N, originating from the doping of N^22^. As a result of the relatively low electronegativity of N (than oxygen), this peak appears at a lower binding energy relative to the C–O and C=O peaks. The N 1s spectra in Fig. 3e show peaks originating from three components, namely graphitic–N, pyrrolic–N, and pyridinic–N^23,24^. The presence of these peaks show that the doping of N atoms into an sp^2^-bonded network of carbon results in three different N functionalities (Fig. 10): (i) graphitic-N (refers to the substitutional N atoms at the carbon sites), (ii) pyrrolic-N (refers to the N atoms that are bonded to two carbon atoms, which contribute to the π system with two p-electrons), and (iii) pyridinic-N (refers to the N atoms at the edge of the graphene planes, each of which is bonded to two carbon atoms and donates one p-electron to the aromatic π–system)^25,26^.

**Figure 4:**
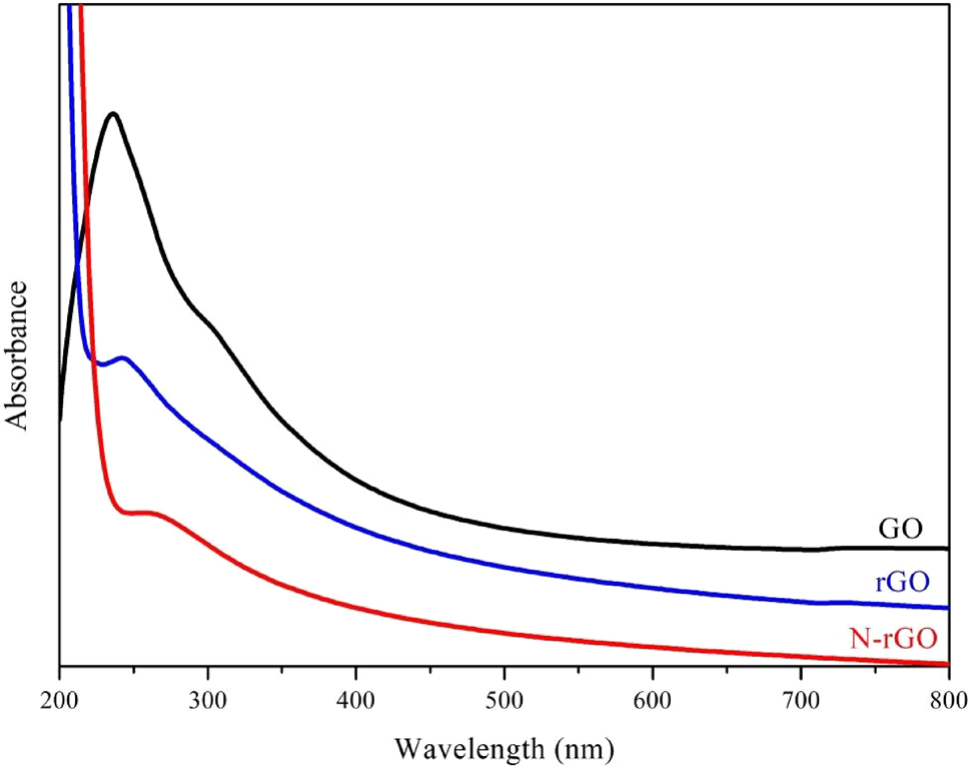
UV–vis absorption spectra of GO, rGO, and N-rGO.

Figure 4 shows the UV–vis absorbance spectra of the GO, rGO, and N-rGO samples. The GO sample exhibited a strong absorbance at 236 and 305 nm, corresponding to π–π* transition of the sp^2^ C=C bonds and n-π* transition of the C=O bonds, respectively^27^. When GO is reduced to graphene, a typical absorption peak is observed at 234 nm, which can be ascribed to the excitation of the π-plasmon of the graphitic structure^28^. The N doping introduces a new UV absorption peak at 268 nm, corresponding to the electron transitions from C=N^29^. Meanwhile, when GO is reduced to rGO and N-rGO, the shoulder peak at approximately 305 nm disappears as a result of the decrease in the concentration of carboxyl groups^30^.

**Figure 5:**
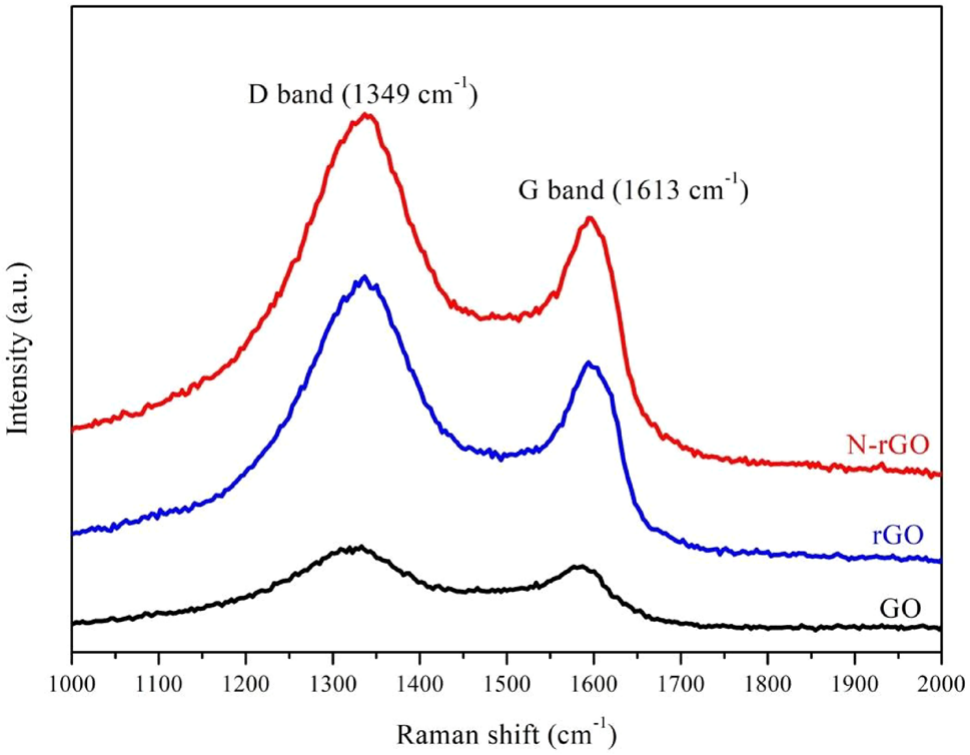
Raman spectra of GO, rGO, and N-rGO.

Figure 5 shows the Raman spectra of the GO, rGO, and N-rGO samples. The D-band, called as a defective band (related to edges, defects, and structurally disordered carbon) is observed at approximately 1349 cm^−1^ and the G-band (in-plane vibrations of sp^2^ carbon network) is observed at approximately 1613 cm^−1^^31^. The intensity ratio of the D-band to the G-band (I_D_/I_G_) is used to determine defect lattices and degree of disorder in graphene^32^. The I_D_/I_G_ ratio increased from 1.15 for GO to 1.24 for rGO. This is a clear indication of the effective reduction of GO. A higher ratio (I_D_/I_G_ = 1.28) for N-rGO indicates the presence of more defects caused by N doping.

### NO sensing characteristics

#### Current–voltage (I–V) characteristics

**Figure 6:**
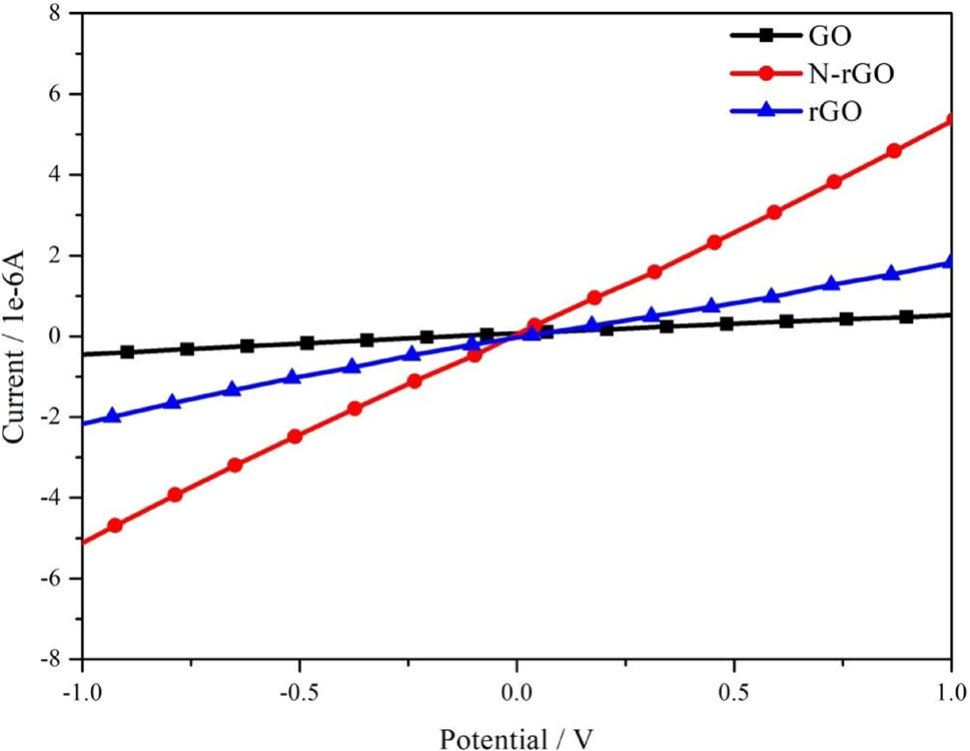
Current–voltage characteristics of GO, rGO, and N-rGO.

Figure 6 shows the I–V characteristics of the NO gas sensors operated at a bias voltage ranging from − 1 to 1 V at room temperature. Graphene oxide is normally electrically insulating at room temperature, owing to the presence of abundant epoxide and hydroxyl groups on both sides and sp^3^-hybridized carbon atoms^33^. After the NaOH treatment, the resistance of rGO slightly decreases, further confirming the reduction of GO. The resistance of N-rGO significantly decreases, indicating improved sensitivity.

#### Reproducibility and stability studies

**Figure 7:**
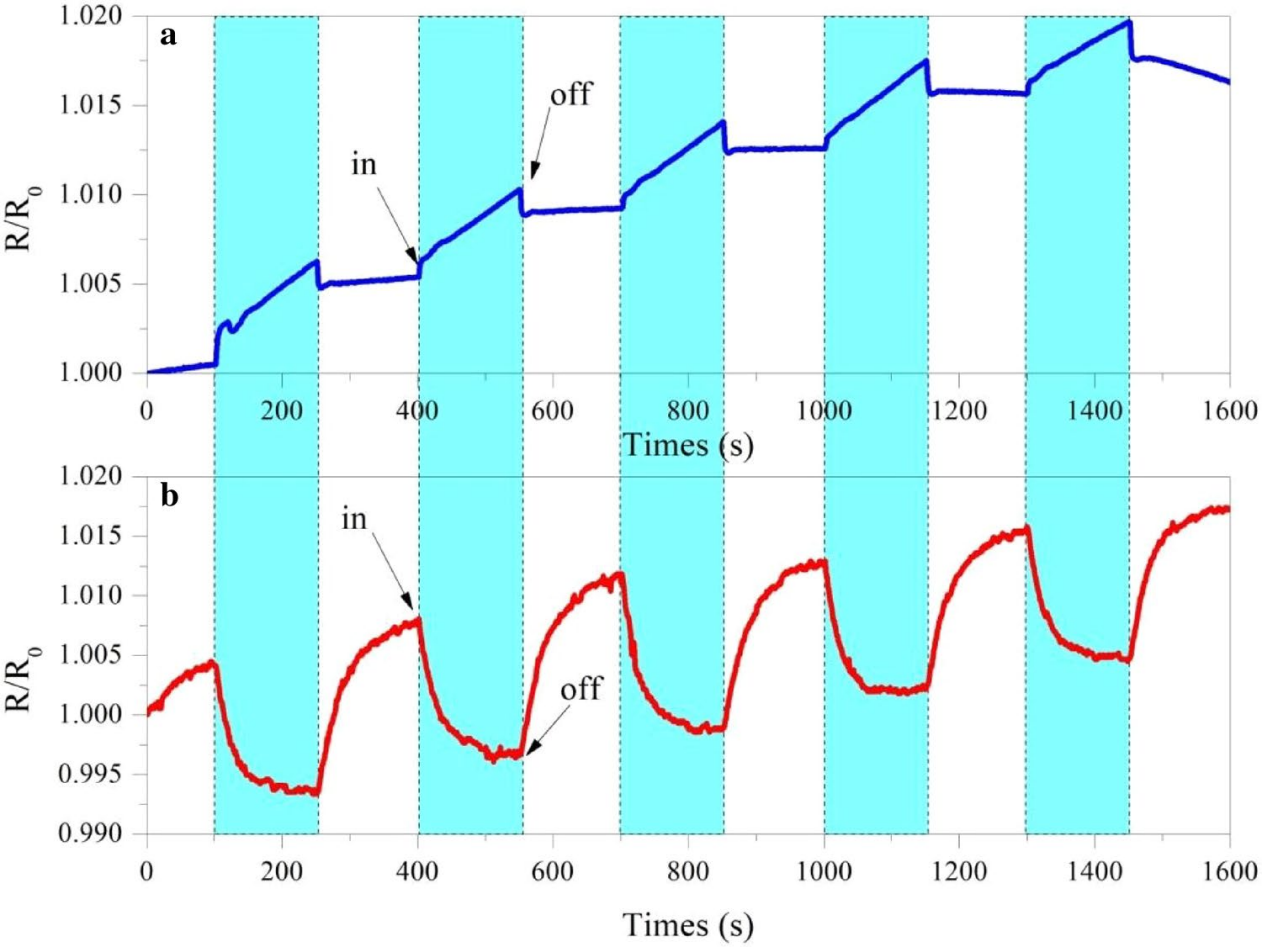
The relative resistance in presence and absence of 1000 ppb NO gas (**a**) N-rGO and (**b**) rGO.

Figure 7 shows the typical dynamic response of the relative resistance (R/R_0_) in 1000-ppb NO at room temperature. Initially, the sensor was exposed to clean air to determine the base resistance; then, NO gas was injected into the sensing chamber for 2.5 min to register the sensing signal; finally, the NO gas supply was closed and air flow was simultaneously allowed for 2.5 min to repeat the cycling of the sensor. In these experiments, there was no evidence of any deterioration in the sensitivity. Figure 7a shows the transport characteristics of the N-rGO device, which is similar to that of an n-type semiconductor. When NO gas is introduced into the test chamber, the NO gas molecules capture the electrons on the N-rGO surface. This reaction leads to a decrease in the concentration of electrons, resulting in a decrease in the conductivity and an increase in the resistance of the sensor^34^. This is because N-rGO is very sensitive to this gas. As the N-rGO nanostructures are exposed to air ambient, the electrons introduced by the oxygen vacancies in the N-rGO nanostructures are adsorbed by the oxygen molecules from the air ambient which compensate the surface oxygen vacancy sites. These surface adsorbed oxygen molecules are transformed into oxygen ions such as O_2_^−^, O_2_^−^ and O^−^ by capturing the electrons from the conduction band of N-rGO. Hence, the electron depletion layer is extended on the surface of the N-rGO nanostructures that increases the electrical resistance of the nanostructures^35^.

 The NO gas is adsorbed on the surface of the N-rGO material according to Eqs. (1), (2), and (3).

1 $$\text{NO(gas)}+{\text{e}}^{-}\rightarrow{\text{NO}}^{-}\text{(ads)}$$

2 $${\text{2NO}}^{-}\text{(ads)}\rightarrow{\text{2O}}^{-}\text{(ads)}+\text{N}_{2}\text{(gas)}$$

3 $${\text{2NO}}^{-}+{\text{e}}^{-}\rightarrow{\text{O}}^{-}\text{(ads)}+\text{N}_{2}\text{O(gas)}$$

**Figure 8:**
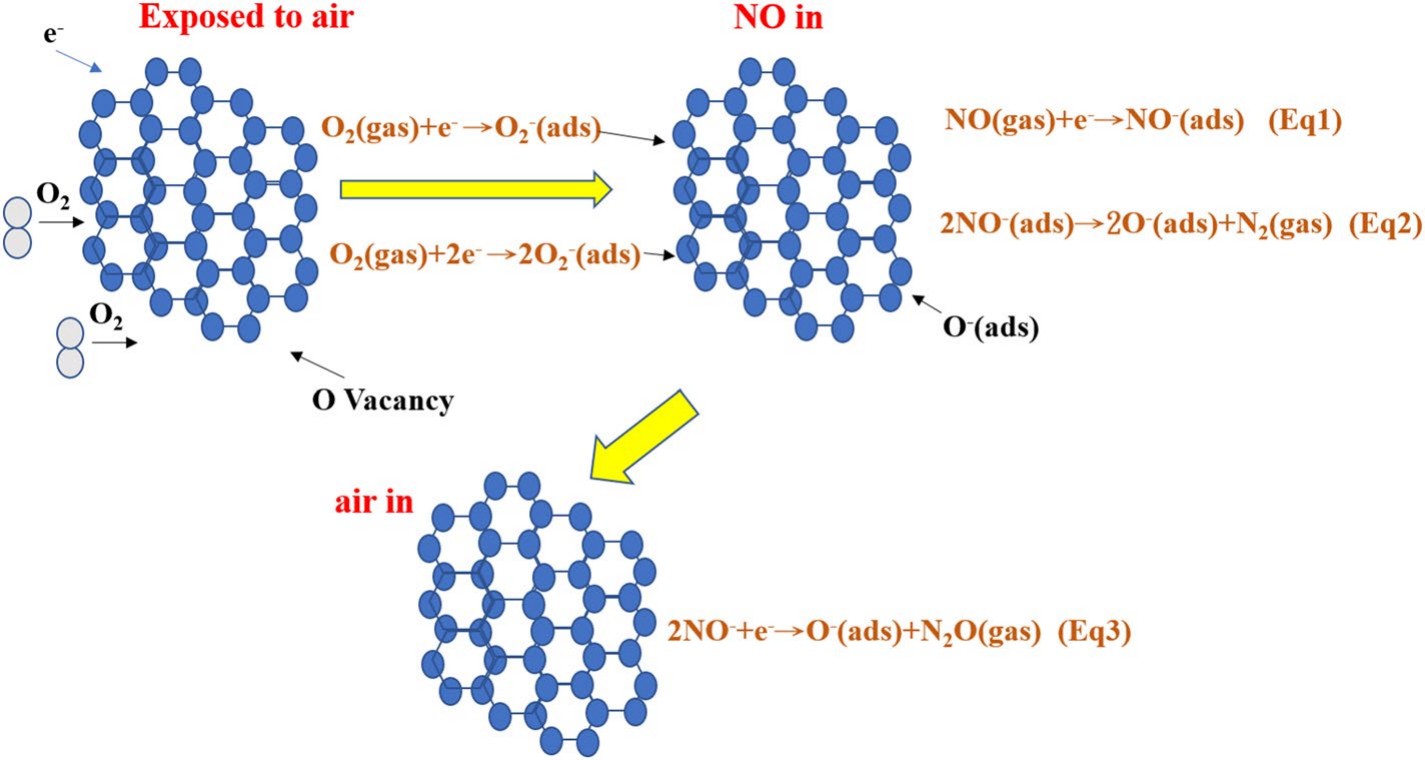
Schematic view of sensing mechanism of N-rGO sensor with NO gas.

Figure 8 shows the N-rGO sensor is exposed to air, oxygen molecules adsorb on the surface of the nanorods form O^−^, O_2_^−^ ions by capturing electrons from the conduction band which results in increase in resistance (Eq. 1). When the N-rGO is exposed to the atmosphere of NO, it captures the electrons due to its higher electrophonic property leading to the formation of adsorbed NO^−^ (ads) which results in increase in resistance (Eq. 2). However, desorption of NO^−^ can take place and the adsorbed NO^−^ (ads) reacts with adsorbed oxygen (O^−^, O_2_^−^) which results in further increase in resistance (Eq. 3). Then, when the air was let in, NO can readily dissociate into O^−^ and some bound electrons were released^36^. Figure 7b shows the transport characteristics of the rGO device, which are similar to those of a p-type semiconductor. This p-type semiconducting characteristic agrees with the results reported for graphene sheets prepared using thermally^37^ and chemically^38^ reduced GO when exposed to the ambient environment. The absorbed oxygen on the rGO surface attracts electrons from graphene, generating additional holes as carriers. This process increases the hole concentration and contributes to the p-type semiconducting behavior. When the rGO (p-type) sample is exposed to the NO gas, a chemical reaction takes place. This chemical reaction will then cause a change in the material properties, for example, electrical conductivity or resistance.

#### Sensitivity studies

**Figure 9:**
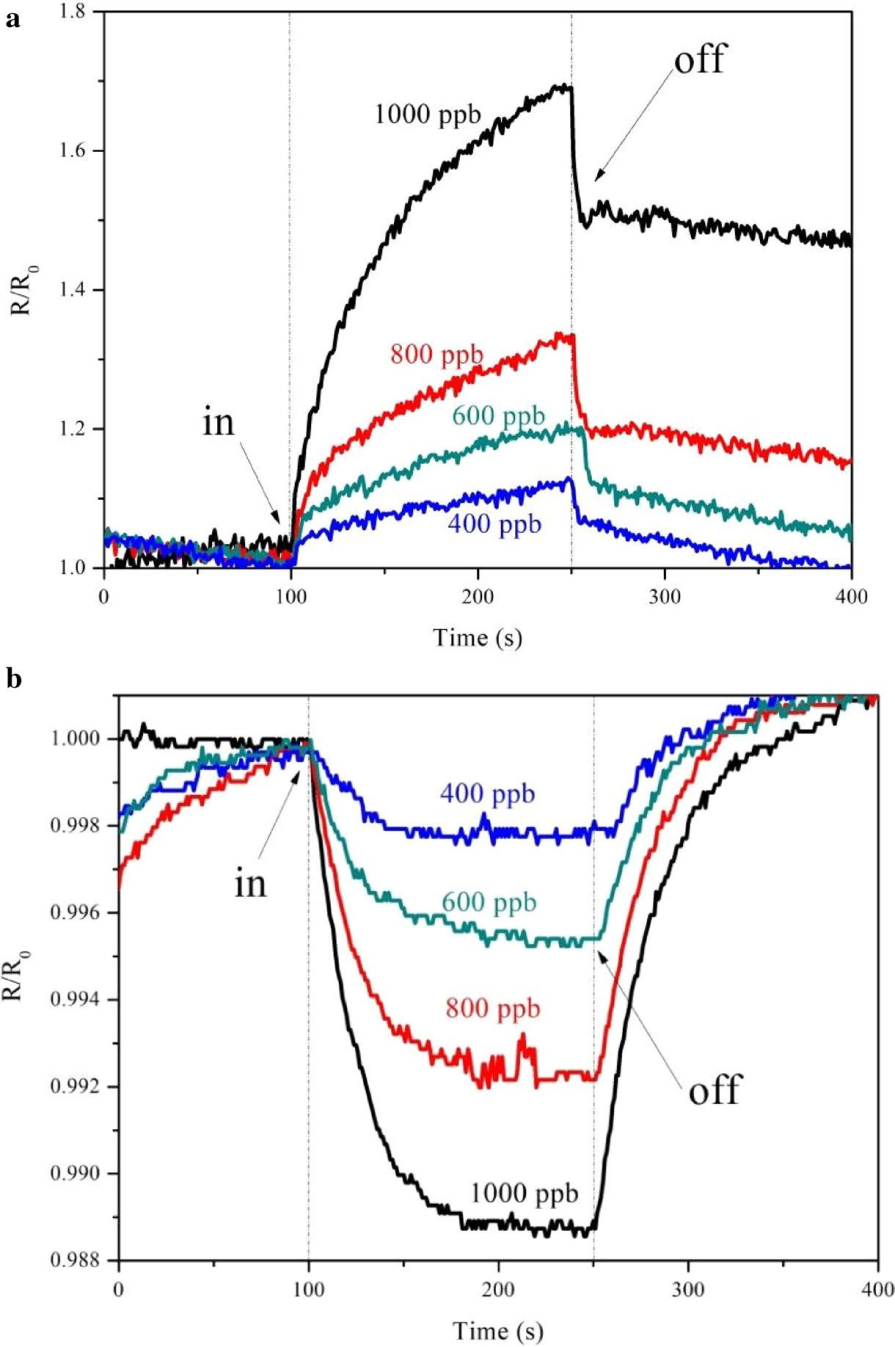
The relative resistance in various concentrations of 1000–400 ppb NO gas (**a**) N-rGO and (**b**) rGO.

**Figure 10:**
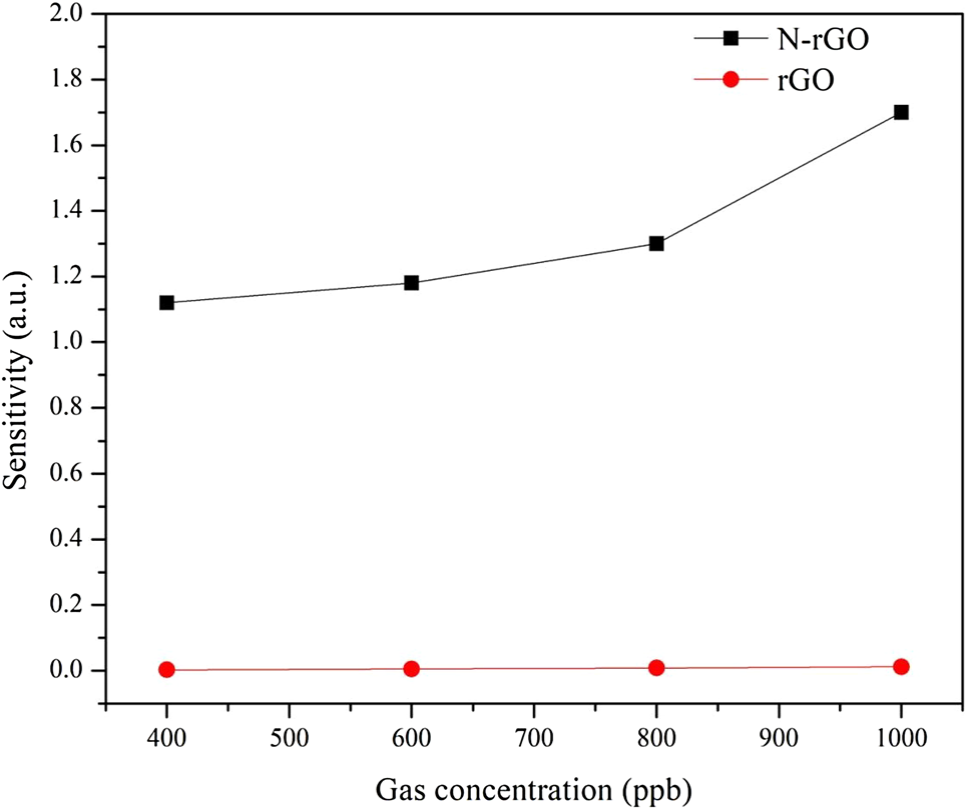
Sensitivity of rGO and N-rGO sensors to NO concentrations ranging from 400 to 1000 ppb at room temperature.

Various concentrations of NO gas, ranging from 400 to 1000 ppb, were fed into the chamber to determine the sensing performance of the optimal sensor. In ambient environments, the oxygen (O_2_) gas excess NO reacts with oxygen to form NO_2_^39^. Therefore, used the N_2_ represent the state the ambient. Figure 9 shows the plots of normalized response versus time for the sensing device based on an assembly of N-rGO and rGO upon exposure to NO gas of different concentrations. The recovery time of the N-rGO sensor was much shorter than that of the rGO sensor, even though sensor recovery was incomplete in N_2_. This means that the surface topography is related to the response time. We calculated the sensitivity (S), which is one of the most important characteristics of a gas sensor^40,41^. Figure 10 shows the relationship curve for the sensitivity to NO at different concentrations ranging from 400 to 1000 ppb. The results indicate that the sensor response increases with the concentration of NO. The sensor sensitivity of rGO is 0.012, 0.008, 0.005, and 0.003, whereas those of N-rGO are 1.7, 1.3, 1.18, and 1.12, that corresponding the NO gas concentrations of 1000 ppb, 800 ppb, 600 ppb, and 400 ppb, respectively. That N-rGO is significantly more sensitive than rGO. In N-doped rGO, the carrier concentration can change the potential profile, which can have a direct influence on the device resistance and the sensing properties.

Table 1 compares the gas-sensing performance attained in the present study with recent results reported in the literature. The use of N-doped rGO sheets greatly improves the sensor performance even in comparison to recently reported results for rGO/metal for the same gases. Thus, it can be concluded that there is a major improvement in the performance of graphene-based gas sensors fabricated with metal- or N-doped graphene.

**Table 1:** Comparison of fabricated NO gas sensors with the previously reported NO sensor material.

References	Sensing material	Working temperature	Concentration	Definition of sensitivity	Response
^42^	rGO/Au	50 °C	5 ppm	R_a_/R_g_	1.15
^13^	rGO/Pt	100 °C	800 ppm	ΔR/R_0_ 100%	12.5%
^15^	rGO	50 °C	5 ppm	R_a_/R_g_	1
This work	rGO	RT	1 ppm	R/R_0_	0.012
	N-rGO	RT	1 ppm	R/R_0_	1.7

#### Sensor performance

**Figure 11:**
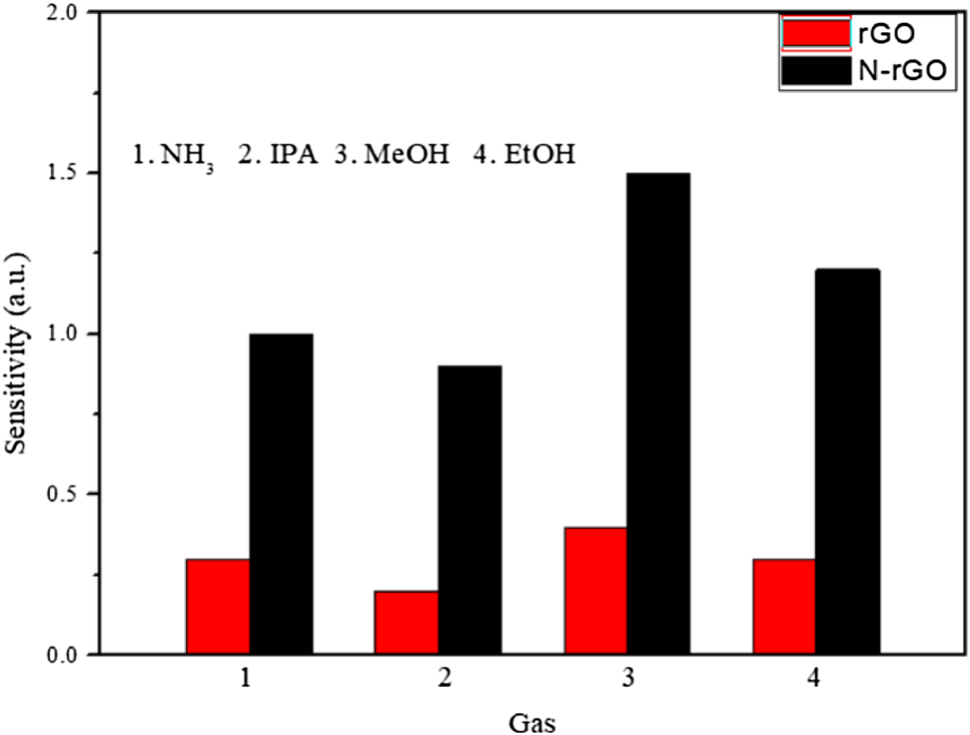
Selectivity of the sensor to other organic volatiles.

Selective detection of target gases remains a challenge in the applications of gas sensors. To identify their selectivity by exposing them to saturation organic vapors like NH_3_, isopropanol (IPA), ethanol (EtOH), methanol (MeOH), as summarized in Fig. 11. It was seen that the responses of the N-rGO sensor are much higher than the rGO sensor on the selective gas.

**Figure 12:**
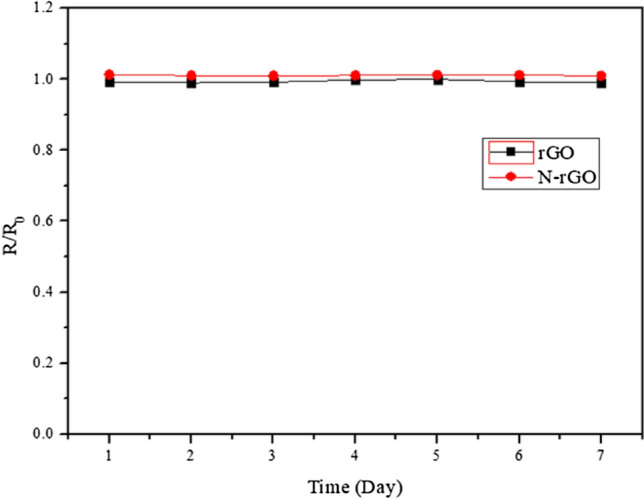
Long-term stability of gas sensor.

The long-term cyclic stability of the rGO and N-rGO sensor was measured in Fig. 12. It can see, the rGO and N-rGO sensors exhibit nearly constant sensing in 7 days at each time to 200 ppm NO during the test. The analysis of humidity is beneficial but we haven't the "Constant temperature and humidity machine". We test the simple for humidity. The corresponding relative humidities of 30%, 50%, and 100% were the NO gas response of 1, 0.7 and 0.2, respectively. This result response decrease, that N-rGO sensors adsorption high water vapor.

### Gas sensing mechanism of N-rGO

**Figure 13:**
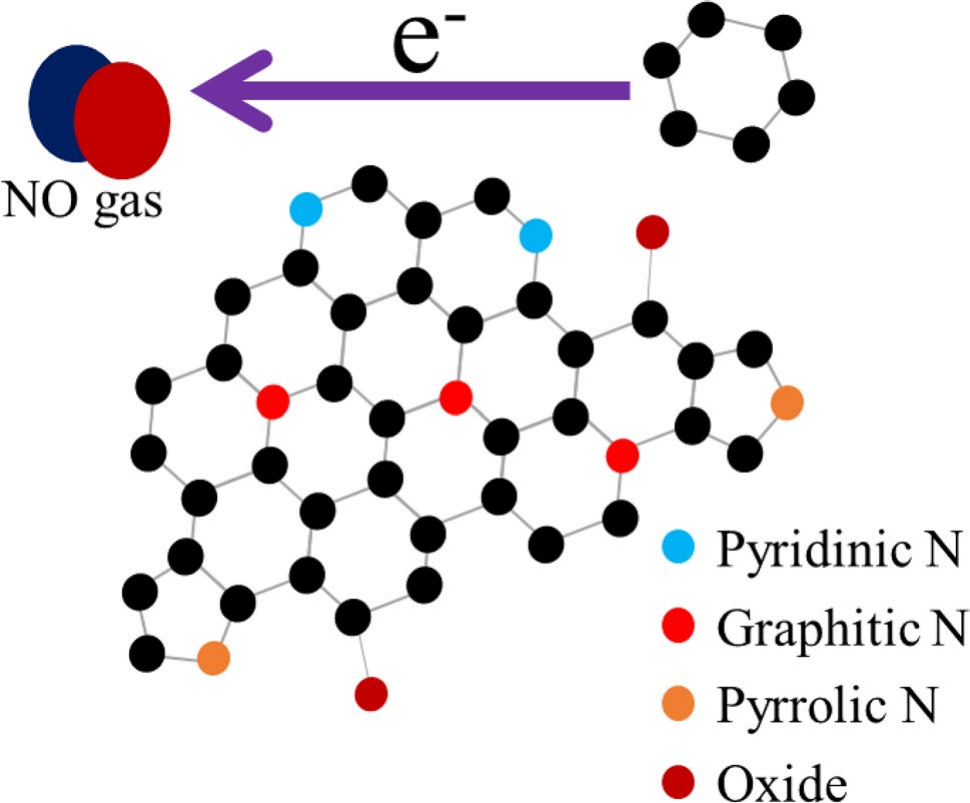
Schematic of the sensing mechanisms of nitric oxide gas adsorbed onto N-rGO sheets.

The gas sensing mechanism of N-rGO can be explained using a surface-controlled model, which includes absorption, electronic desorption, and transfer. The unsatisfactory performance of the rGO sensor results from its constituent carbon atoms. Graphene has two types of carbon atoms, namely sp^2^ hybridized carbon atoms and sp^3^ hybridized carbon atoms. These constitute the graphite structure and the structural defects, and form chemical bonds with oxygen-containing groups, respectively. The absorption energy of sp^3^ is larger (5.7 kcal/mol), resulting in slower adsorption and desorption^43^. Figure 13 shows the schematic of the sensing mechanism of NO gas adsorbed onto N-rGO sheets. The improvement in the NO gas-sensing performance of the rGO nanosheets doped with N can be explained as follows: (i) Gas sensing by resistance-type sensors is based on variations in the conductance of the sensing element. The N-doped rGO offers significantly improved sensitivity, leading to a better sensing behavior; (ii) The N doping would produce polarization in the sp^2^ carbon network, in which N has a higher electronegativity (x = 3.04) than C (x = 2.55), thus further affecting the physical and the chemical performance; (iii) The doped N atoms exist in three different states, namely pyridinic-N, graphitic-N, and pyrrolic-N. The enhanced catalytic activity is usually attributed to the increased active sites of pyridinic-N and/or pyrrolic-N. Pyridinic N refers to N atoms at the edges of graphene planes, where each N atom is bonded to two carbon atoms and donates one p-electron to the aromatic π system^44,45^. Pyrrolic N atoms are incorporated into five-membered heterocyclic rings, which are bonded to two carbon atoms and contribute two p-electrons to the π system^46–48^. Pyridinic-N modifies the band structure of carbon, raising the density of π states near the Fermi level and lowering the work function.the relative electronegativity of graphitic N atoms reduces the electron density on the adjacent C nuclei, which helps electrons transfer from the adjacent C to N atoms, and N backdonates electrons to adjacent C pz orbitals. The donation and backdonation processes not only facilitate O_2_ dissociation on the adjacent C atoms, but also help forming a strong chemical bond between O and C^49^.

## Conclusions

Reduced graphene oxide and N-rGO nanosheets were evaluated for their NO gas sensing properties at room temperature. The hydrothermal treatment converted rGO to n-type N-rGO, as confirmed by UV and XPS analyses. The gas-sensing results showed that the N-rGO sensors could detect NO gas at concentrations as low as 400 ppb. The sensitivity of the N-rGO sensor to 1000 ppb NO (1.7) is much better than that of the rGO sensor (0.012). The results of the present study indicate that N-doping can significantly enhance the NO sensing properties of graphene-based sensing materials at room temperature, suggesting their excellent potential for use as gas sensors.

## References

[1] Galstyan V (2017). Porous TiO_2_-based gas sensors for cyber chemical systems to provide security and medical diagnosis. Sensors.

[2] Liu X, Cheng S, Liu H, Hu S, Zhang D, Ning H (2012). A survey on gas sensing technology. Sensors.

[3] Pandey S (2016). Highly sensitive and selective chemiresistor gas/vapor sensors based on polyaniline nanocomposite: A comprehensive review. J. Sci. Adv. Mater..

[4] Pandey S, Ramontja J (2016). Rapid, facile microwave-assisted synthesis of xanthan gum grafted polyaniline for chemical sensor. Int. J. Biol. Macromol..

[5] Kumar R, Al-Dossary O, Kumar G, Umar A (2015). Zinc oxide nanostructures for NO_2_ Gas-Sensor applications: A review. Nano Micro Lett..

[6] Dey A (2018). Semiconductor metal oxide gas sensors: A review. Mater. Sci. Eng. B.

[7] Kawano T, Chiamori HC, Suter M, Zhou Q, Sosnowchik BD, Lin L (2007). An electrothermal carbon nanotube gas sensor. Nano Lett..

[8] Zanolli Z, Leghrib R, Felten A, Pireaux J-J, Llobet E, Charlier J-C (2011). Gas sensing with Au-decorated carbon nanotubes. ACS Nano.

[9] Akbari E, Buntat Z, Ahmad MH, Enzevaee A, Yousof R, Iqbal SMZ, Ahmadi MT, Sidik MAB, Karimi H (2014). Analytical calculation of sensing parameters on carbon nanotube based gas sensors. Sensors.

[10] Bolotin KI, Sikes KJ, Jiang Z, Klima M, Fudenberg G, Hone J, Kim P, Stormer HL (2008). Ultrahigh electron mobility in suspended grapheme. Solid State Commun..

[11] Bernal MM, Tortello M, Colonna S, Saracco G, Fina A (2017). Thermally and electrically conductive nanopapers from reduced graphene oxide: Effect of nanoflakes thermal annealing on the film structure and properties. Nanomaterials.

[12] Lu G, Ocola LE, Chen J (2009). Reduced graphene oxide for room-temperature gas sensors. Nanotechnology.

[13] Wang J, Rathi S, Singh B, Lee I, Maeng S, Joh H-I, Kim G-H (2015). Dielectrophoretic assembly of Pt nanoparticle-reduced grapheme oxide nanohybrid for highly-sensitive multiple gas sensor. Sens. Actuators B Chem..

[14] Galstyan V, Comini E, Kholmanov I, Faglia G, Sberveglieri G (2016). Reduced graphene oxide/ZnO nanocomposite for application in chemical gas sensors. RSC Adv..

[15] Zhang H, Wang LL, Zhang T (2014). Reduced graphite oxide/SnO_2_/Au hybrid nanomaterials for NO_2_ sensing performance at relatively low operating temperature. RSC Adv..

[16] Liu S, Wang Z, Zhang Y, Dong Z, Zhang T (2015). Preparation of zinc oxide nanoparticle–reduced graphene oxide–gold nanoparticle hybrids for detection of NO_2_. RSC Adv..

[17] Marcano DC, Kosynkin DV, Berlin JM, Sinitskii A, Sun Z, Slesarev A, Alemany LB, Lu W, Tour JM (2010). Improved synthesis of graphene oxide. ACS Nano.

[18] Wu J, Tao K, Miao J, Norford LK (2015). Improved selectivity and sensitivity of gas sensing using a 3D reduced graphene oxide hydrogel with an integrated microheater. ACS Appl. Mater. Interfaces.

[19] Jeong SY, Jeong S, Lee SW, Kim ST, Kim D, Jeong HJ, Han JT, Baeg K-J, Yang S, Jeong MS, Lee G-W (2015). Enhanced response and sensitivity of self-corrugated graphene sensors with anisotropic charge distribution. Sci. Rep..

[20] Fu C, Zhao G, Zhang H, Li S (2013). Evaluation and characterization of reduced graphene oxide nanosheets as anode materials for lithium-ion batteries. Int. J. Electrochem. Sci..

[21] Muralikrishna S, Sureshkumar K, Varley TS, Nagaraju DH, Ramakrishnappa T (2014). In situ reduction and functionalization of graphene oxide with l-cysteine for simultaneous electrochemical determination of cadmium(II), lead(II), copper(II), and mercury(II) ions. Anal. Methods.

[22] Nolan H, Sanchez BM, Kumar NA, Mcevoy N, Obrien S, Nicolosi V, Duesberg GS (2014). Nitrogen-doped reduced graphene oxide electrodes for electrochemical supercapacitors. Phys. Chem. Chem. Phys..

[23] Vinoth R, Babu SG, Bahnemann D, Neppolian B (2015). Nitrogen doped reduced graphene oxide hybrid metal free catalysts for effective reduction of 4-nitrophenol. Sci. Adv. Mater..

[24] Li S, Miao H, Xu Q, Xue Y, Sun S, Wang Q, Liu Z (2016). Silver nanoparticles supported on a nitrogen doped graphene aerogel composite catalyst for an oxygen reduction reaction in aluminum air batteries. RSC Adv..

[25] Liu S, Xie J, Li H, Wang Y, Yang HY, Zhu T, Zhang S, Cao G, Zhao X (2014). Nitrogen-doped reduced graphene oxide for high performance flexible all-solid-state microsupercapacitors. J. Mater. Chem. A..

[26] Boutchich M, Arezki H, Alamarguy D, Ho K-I, Sediri H, Gunes F, Alvarez J, Kleider JP, Lai CS, Ouerghi A (2014). Atmospheric pressure route to epitaxial nitrogen-doped trilayer graphene on 4H-SiC (0001) substrate. Appl. Phys. Lett..

[27] Singhbabu YN, Sahu KK, Dadhich D, Pramanick AK, Mishra T, Sahu RK (2013). Capsule-embedded reduced graphene oxide: Synthesis, mechanism and electrical properties. J. Mater. Chem. C..

[28] Yang Y, Yang X, Yang W, Li S, Xu J, Jiang Y (2014). Porous conducting polymer and reduced graphene oxide nanocomposites for room temperature gas detection. RSC Adv..

[29] Hu C, Liu Y, Yang Y, Cui J, Huang Z, Wang Y, Yang L, Wang H, Xiao Y, Rong J (2013). One-step preparation of nitrogen-doped graphene quantum dots from oxidized debris of graphene oxide. J. Mater. Chem. B..

[30] Zhang Y, Ma H-L, Zhang Q, Peng J, Li J, Zhai M, Yu Z-Z (2012). Facile synthesis of well-dispersed graphene by g-ray induced reduction of graphene oxide. J. Mater. Chem..

[31] Gong Y, Li D, Fu Q, Pan C (2015). Influence of graphene microstructures on electrochemical performance for supercapacitors. Prog. Nat. Sci. Mat. Int..

[32] Zuo X, Li B, Chang K, Tang H, Chang Z (2017). Tin-based materials supported on nitrogen-doped reduced graphene oxide towards their application in lithium-ion batteries. RSC Adv..

[33] Sarkar SK, Raul KK, Pradhan SS, Basu S, Nayak A (2014). Magnetic properties of graphite oxide and reduced graphene oxide. Phys. E.

[34] Yuliarto B, Kumai Y, Inagaki S, Zhou H (2009). Enhanced benzene selectivity of mesoporous silica SPV sensors by incorporating phenylene groups in the silica framework. Sens. Actuators B Chem..

[35] Venkateshab PS, Dharmaraja P, Purushothamanac V, Ramakrishnand V, Jeganathan K (2015). Point defects assisted NH_3_ gas sensing properties in ZnO nanostructures. Sens. Actuators B.

[36] Zou C, Liang F, Xue S (2015). Synthesis and oxygen vacancy related NO_2_ gas sensing properties of ZnO: Co nanorods arrays gown by a hydrothermal method. Appl. Surf. Sci..

[37] Jung I, Dikin DA, Piner RD, Ruoff RS (2008). Tunable electrical conductivity of individual graphene oxide sheets reduced at “Low” temperatures. Nano Lett..

[38] Gilje S, Han S, Wang M, Wang KL, Kaner RB (2007). A chemical route to graphene for device applications. Nano Lett..

[39] Yuliarto B, Zhou HS, Yamade T, Honma I, Katsumura Y, Ichihara M (2014). Effect of Tin addition on mesoporous silica thin film and its application for surface photovoltage NO_2_ gas sensor. Anal. Chem..

[40] Mao S, Cui S, Lu G, Yu K, Wen Z, Chen J (2012). Tuning gas-sensing properties of reduced graphene oxide using tin oxide nanocrystals. J. Mater. Chem..

[41] Zhou Y, Xie GZ, Xie T, Yuan H, Tai HL, Jiang YD, Chen Z (2014). A sensitive film structure improvement of reduced graphene oxide based resistive gas sensors. Appl. Phys. Lett..

[42] Zhang H, Li Q, Huang J, Du Y, Ruan SC (2016). Reduced graphene oxide/Au nanocomposite for NO_2_ sensing at low operating temperature. Sensors.

[43] Fowler JD, Allen MJ, Tung VC, Yang Y, Kaner RB, Weiller BH (2009). Practical chemical sensors from chemically derived graphene. ACS Nano.

[44] Chen Z, Higgins D, Chen Z (2010). Nitrogen doped carbon nanotubes and their impacton the oxygen reduction reaction in fuel cells. Carbon.

[45] Biddinger EJ, Ozkan US (2010). Role of graphitic edge plane exposure in carbon nanostructures for oxygen reduction reaction. J. Phys. Chem. C.

[46] Kurak KA, Anderson AB (2009). Nitrogen-treated graphite and oxygen electroreduction on pyridinic edge sites. J. Phys. Chem. C.

[47] Lee KR, Lee KU, Lee JW, Ahn BT, Woo SI (2010). Electrochemical oxygen reduction on nitrogen doped graphene sheets in acid media. Electrochem. Commun..

[48] Luo Z, Lim S, Tian Z, Shang J, Lai L, MacDonald B, Fu C, Shen Z, Yu T, Lin J (2011). Pyridinic N doped graphene: Synthesis, electronic structure, and electrocatalytic property. J. Mater. Chem..

[49] Deng D, Pan X, Yu L, Cui Y, Jiang Y, Qi J, Li WX, Fu Q, Ma X, Xue Q, Sun G (2011). Toward N-doped graphene via solvothermal synthesis. Chem. Mater..

